# Reducing inequities in maternal and child health in rural Guatemala through the CBIO+ Approach of Curamericas: 3. Expansion of population coverage of key interventions

**DOI:** 10.1186/s12939-022-01755-9

**Published:** 2023-02-28

**Authors:** Stanley Blanco, Mario Valdez, Ira Stollak, Carey C. Westgate, Andrew Herrera, Henry B. Perry

**Affiliations:** 1 Consejo de Salud Rural Andino/Curamericas, La Paz, Bolivia; 2 Curamericas/Guatemala, Calhuitz, San Sebastián Coatán, Huehuetenango, Guatemala; 3 Curamericas Global, Raleigh, North Carolina USA; 4 Community Health Impact Coalition, New York, New York USA; 5 Health Systems Program, Department of International Health, Johns Hopkins Bloomberg School of Public Health, Baltimore, Maryland USA

**Keywords:** Maternal health, Child health, Community health, Primary health care, Community-based primary health care, Implementation research, Census-Based, Impact-Oriented Approach, Care Groups, Community birthing centers, Guatemala, Equity, Curamericas Global, Curamericas/Guatemala

## Abstract

**Background:**

This is the third in a series of 10 articles describing the Curamericas/Guatemala Maternal and Child Health Project, 2011–2015, and its effectiveness in improving the health and well-being of 15,327 children younger than 5 years of age and 32,330 women of reproductive age in the Department of Huehuetenango in180 communities that make up the municipalities of San Sebastian Coatán, Santa Eulalia, and San Miguel Acatán. The Project combined the Census-Based, Impact-Oriented (CBIO) Approach with the Care Group Approach and the  Community Birthing Center (*Casa Materna Rural*) Approach. This combined approach we refer to as CBIO+. The Project trained women volunteers every two weeks (in Care Groups) to provide health education to neighboring households. Messages focused on the promotion of maternal and newborn health, nutrition, prevention and treatment of acute respiratory infection and diarrhea in children, and immunizations.

**Methods:**

Household knowledge, practice and coverage (KPC) surveys were executed at baseline in January 2011 and at endline in June 2015 to measure changes in levels of knowledge of danger signs, key household practices (such as Essential Newborn Care and handwashing), and health service utilization (such as antenatal care and care seeking for a child with signs of pneumonia) in two separate Project Areas (Area A with 41 months and Area B with 20 months of full intervention implementation).

**Results:**

For the 24 indicators of the interventions under the Project’s control, statistically significant improvements were observed for 21 in Area A and 19 in Area B. However, for some of the interventions that required support from the government's Extension of Coverage Program (immunization, family planning, and vitamin A administration) no improvements were noted because of the cessation of the program by the government after Project implementation began. In both Areas A and B one-half of the indicators improved by at least two-fold.

**Conclusion:**

This community-based Project has been effective in quickly achieving marked improvements in indicators for interventions that are important for the health of mothers and children. These achievements are notable in view of the challenging context in which the Project was implemented.

## Background

Between 2011 and 2015, Curamericas/Guatemala implemented the Maternal and Child Health Project (hereafter referred to as the Project) in the entire municipalities^1^ of Sebastián Coatán, San Miguel Acatán, and Santa Eulalia, which have a combined population of 98,000 people. The goal of the Project was to improve the health and well-being of mothers and children through an empowering participatory process known as CBIO+. CBIO refers to the Census-Based, Impact-Oriented Approach: the “+” refers to the inclusion of Care Groups and Community Birthing Centers (called *Casas Maternas Rurales)*. CBIO+ was integrated into the existing health services of the Guatemala Ministry of Health (*Ministerio de Salud Pública y Asistencia Social/*MSPAS). Table 1 provides a brief overview of CBIO+. Additionally, the Project integrated its efforts with MSPAS Extension of Coverage Program (*Programa de Extensión de Cobertura,* or PEC).

**Table 1:** CBIO+ explained

The CBIO+ Approach is an expansion of CBIO. It is composed of three components: (1) the Census-Based, Impact-Oriented (CBIO) Approach, (2) the Care Group Approach, and (3) the Community Birthing Center Approach. CBIO consists of conducting a house-to-house census, registering all households, identifying local epidemiological priorities and the health priorities according to the local people, developing a plan to address these priorities, and assessing over time whether the health of the population has improved [1]. All of this is accomplished through partnerships with the community, collection of local data, and routine systematic home visitation to collect data, including vital events, and to deliver services. Further descriptions of the CBIO approach and its effectiveness are available [2–6]. The Care Group Approach is, in a sense, an extension of CBIO that involves the selection of one female Care Group Volunteer for every 10-15 households. Then, 5-12 Care Group Volunteers meet with a Care Group Promoter every 2–4 weeks to learn 1–2 educational messages to share, either by visiting each home separately or meeting as a group, with the mothers in the catchment area for each Care Group Volunteer. At the subsequent meeting, the Promoter teaches them a new message and the Care Group Volunteers report pregnancies, births and deaths to the Promoter [7]. Further descriptions of the Care Group Approach and its effectiveness are available [8–11]. The Community Birthing Center Approach as developed by Curamericas/Guatemala is a participatory approach that involves working with communities to construct, staff and operate a readily available local facility where mothers can give birth in a way that respects traditional customs and enables the traditional midwife (called a *comadrona* in the Project Area) to perform her traditional role. These centers are staffed 24/7 by auxiliary nurses with special additional training in midwifery and supervised by an experienced obstetrical graduate nurse who is based at one of the Birthing Centers and is available in person or by phone to support the auxiliary nurses in all the Centers should the need arise. Connected to each Birthing Center is an emergency transport system to provide quick referral to a hospital should the need arise. Also associated with the Birthing Center is an insurance system that pregnant women and their families can pay into during the pregnancy to offset to cost of transport if a referral is needed. Further descriptions of the Community Birthing Center Approach are available [12].	

These communities are located in the Cuchumatanes Mountains, an isolated mountain region in the western highlands of the Guatemalan altiplano where the population is overwhelmingly Maya. This indigenous population exhibits some of the worst health indicators in Latin America; hence, the name locally given to this region is “the Triangle of Death,” arising initially from the persecution of indigenous people in this area during the Guatemala Civil War (1960–1996) [13].

This article is the third in a series of papers that summarize the findings of an evaluation of the Project. The first article provides an introduction to the Project as well as a detailed description of CBIO, Care Groups, and Community Birthing Centers (*Casas Maternas Rurales*) and their implementation [14]. The second article describes the implementation research strategy [15]. In this article we describe changes in the population coverage of key interventions for maternal and child health including knowledge, household behaviors, and utilization of health services. The fourth through tenth articles focus on nutrition [16], mortality [17], quality of care provided at Community Birthing Centers [18], women’s empowerment [19, 20], assessment of CBIO+ by key stakeholders [21], and cost-effectiveness and policy implications [22].

### Project description, approach and interventions

As described more fully in Papers 1 and 2 [14, 15], the Project Area contained 15,327 children younger than 5 years of age and 32,330 women of reproductive age. Interventions focused on peer-to-peer health education in the home to promote maternal/newborn health and nutrition, prevention and treatment of acute respiratory infections and diarrhea, and immunizations. The Project also aimed to respond to local health priorities and challenges by integrating its efforts with the services of the MSPAS to create a coherent local rural health system that addressed community and epidemiological priorities, integrating the CBIO+ approach with the MSPAS Extension of Coverage Program (PEC). PEC provided a mobile health team of nurses that visited villages monthly to provide immunizations, nutritional monitoring, micronutrient supplementation, family planning services, and community case management of childhood illnesses.

The principal intervention strategies of the Project included:

- Mobilize communities and promote household behaviors and health service utilization that reduce morbidity and mortality in mothers and children. This was achieved by working in partnership with communities to make a census of all households, establish Care Groups of 5–12 volunteers who met with a Care Group Promoter every 2 weeks to learn health education messages, and then convey these messages through home visits and neighborhood group meetings for 10–15 mothers in their catchment area.
- Improve the capacity of local partners, health facilities, and health workers to provide quality information and services for the continuum of care for mothers and children. This was achieved through training and support for all health workers in the Project Area, including *comadronas* (traditional midwives).
- Establish emergency response transport networks to address obstetric, neonatal, and child emergencies. This was achieved by working with the communities that supported Community Birthing Centers to arrange an insurance system for paying for transport when obstetric emergencies arose there, but the system was also used for other medical emergencies.
- Increase utilization of and access to high-impact interventions for pregnant women, new mothers, and children younger than 5 years of age. This was achieved by raising awareness about the importance of key interventions, promoting healthy household behaviors (including Essential Newborn Care, appropriate infant and young child feeding, prevention and treatment of childhood diarrhea, handwashing and household hygiene), and utilization of health services for antenatal care and delivery and when symptoms of childhood pneumonia develop.

As described in greater detail in Paper 1 [14], the Project was implemented in two areas. Area A encompassed 89 communities where services were provided beginning in the first two years of the Project (PY1 and PY2). During Years 3 and 4 of the Project, services continued in Area A while the same services began in Area B, with 91 communities. This design made it possible during the first two years to (1) perform formative research designed to derive lessons learned from implementing CBIO+ in Area A for application in Area B, (2) compare the difference between the endline results of Areas A and B to determine if there was a dose-response effect after four years of implementation in Area A compared to two years in Area B, and (3) compare endline results in Areas A and B with coverage levels in comparison areas outside of the Project Area.

## Methods

Paper 2 provides detailed information about the implementation research methods used [15]. Here we provide additional information about the household surveys carried out.

### Survey design and strategy

At the start of the Project in late 2011 and early 2012, a baseline knowledge, practice, and coverage (KPC) survey was designed and carried out in Areas A and B separately to measure population coverage of Project interventions and levels of knowledge, key behaviors, and utilization of health services. At the end of the Project implementation period in 2015, the same KPC methodology and survey instruments were used to measure endline coverage of the same indicators.

The survey design involved consultations with external evaluators and in-country staff to determine the sampling strategy, develop the actual sample, and assess all data needs. Next, the evaluation team finalized the survey questionnaire and planned the logistics, strategy, and training of supervisors and interviewers. The survey implementation involved recruiting, selecting, and training data collectors who were responsible for the actual data collection in all selected communities of the three municipalities. The data were analyzed using Epi Info 7.1. Manual data analysis was also performed using MS Excel.

The baseline surveys for Areas A and B were conducted in January 2012 and the final surveys in May/June of 2015. The endline assessment was designed to be participatory, engaging Project staff, stakeholders, partners, and beneficiaries, including Curamericas/Guatemala staff, representatives of local municipalities, partners, community authorities, municipal and departmental representatives of MSPAS, as well as target beneficiary families. During the training of interviewers, community leaders helped interviewers with oral translation into the indigenous Maya languages and, in addition, an MSPAS official participated actively together in the Epi Info data management training of the data entry staff.

Nearly identical survey questionnaires were used for the baseline and final surveys. The survey questionnaire was written in Guatemalan Spanish with language reviewed and approved by the Curamericas/Guatemala staff. A copy of the questionnaire is available from the corresponding author on request.

The indicators are standard maternal and child health indicators used in demographic and health surveys throughout the world [23] and by the United States Agency for International Development (USAID) Child Survival and Health Grants Program [24]. Appendix 1 contains the full definition for each indicator in the analysis. Information about antenatal care and maternal tetanus toxoid immunization were obtained from the maternal health card when possible, and information about childhood immunization was obtained from the child health card when possible. “Quality antenatal care” was defined as a prenatal visit with a trained healthcare provider (doctor, nurse, or nurse-midwife). In order to determine if there was active management of the third stage of labor (AMTSL), the respondent was asked if the birth attendant massaged her uterus after delivery, if the attendant pulled on the umbilical to extract the placenta, and if the mother received an injection immediately after the delivery. (There was no misoprostol used in the Project Area at that time, so an injection would imply the use of oxytocin.) Essential Newborn Care was defined as providing immediate breastfeeding (within 1 h after birth), drying and wrapping the newborn immediately after birth, and cutting the umbilical with a sterile instrument.

Appropriate care seeking for a child with symptoms of pneumonia required the mother reporting that she took her child with difficult or rapid breathing to a trained healthcare worker (doctor, nurse, or auxiliary nurse) within 48 hours of the onset of these symptoms. Knowledge of handwashing was present if the respondent could spontaneously name the four critical times when a mother should wash her hands (after defecating, after cleaning a child who had defecated, before preparing food, and before feeding a child).

### Sampling

The baseline and final KPC surveys used the same 300-respondent stratified cluster sampling technique described in the second paper of this supplement [15]. Two sampling frames were utilized, one for each of the two Project Areas, Area A and Area B. Thirty clusters were randomly selected from each Area with probability proportional to size, and 10 nearby households were surveyed in each cluster after first randomly selecting one household in the cluster. Based on standard sampling guidelines for KPC surveys from USAID and the CORE Group using stratified cluster sampling [24], a sample size of 300 with a design effect of 2.0 and a 95% confidence interval of +/− 8%.

### Selection and training of supervisors, interviewers and tabulators

For the Baseline KPC Survey, 40 interviewers were hired who were not Project staff. These were persons with at least a high school diploma who spoke the Maya dialect in the area where they would interview. They were supervised by 12 field supervisors who were Curamericas/Guatemala staff and who were also native speakers of the local dialect. All field supervisors and interviewers were female. Their work was overseen by three municipal supervisors, all Curamericas/Guatemala staff.

For the Final KPC Survey, the Curamericas/Guatemala team used 20 existing staff as field supervisors of interviewers. They were familiar with the Project’s objectives, the KPC methodology, and the Project Area. The Project hired additional persons who were conversant in the local Maya dialect to serve as interviewers. Some Curamericas/Guatemala staff members also served as interviewers for the final survey; they were all conversant in the local Maya dialect and interviewed in communities where they had not worked previously in Project implementation activities. There were 34 interviewers in total. The field supervisors and interviewers were all female. Each of the three municipalities had an overall municipal supervisor responsible for the KPC data collection in his/her municipality; each municipal supervisor supported 5-9 field supervisors and 10-14 interviewers.

Training sessions were conducted in Spanish by one of the authors (IS for the baseline survey and SB for the endline survey) at the Curamericas/Guatemala headquarters in Calhuitz, San Sebastian Coatán. The interviewers, supervisors, and data technicians learned the purpose of the Project, acquired an understanding of the Project indicators and corresponding KPC questions, and practiced both in the workshop and in the field (in communities not included in the sample).

The training included workshops in which the interviewers and supervisors reached consensus on the proper translation of interview questions and answer options from Spanish to the Maya language (Chuj, Akateko, or Q’anjob’al). The supervisors and interviewers were native speakers of the Maya language of the households in their assigned clusters. This involved lengthy discussions. After field-testing the questionnaire, they repeated the process, focusing on questions for which there were still translation issues and reaching a new consensus on those questions. Training included a full day in the field. Interviewers each conducted four to five interviews – including weighing and measuring the length of children. These were observed and evaluated by a field supervisor. Supervisors utilized a quality control checklist to ensure that the necessary skills were acquired before the interviewers were allowed to begin the actual survey. Improvements in translation were made incorporating the preliminary field work experience.

### Survey process and quality control

The supervisors were responsible for directly observing a sample of the interviews. They reviewed every completed questionnaire for completeness and accuracy and then signed their approval for each one. The completed questionnaires were then packed in envelopes and sent to the Project headquarters in Calhuitz for data entry by the data technicians. Data entry staff reviewed the submitted questionnaires for completeness and for clarity of responses. If problems were detected, the questionnaire was returned to the field supervisor with written observations and recommendations for correction.

### Tabulation, data entry, and quality control

For the Baseline KPC Survey, Excel spreadsheets were used to tabulate and analyze baseline survey results and generate values and confidence intervals for each result from each Area. Excel data entry was cross-checked by the data technicians, who were trained Curamericas/Guatemala staff. A year later, the baseline Excel dataset was entered into Epi Info 7 by a graduate student intern and the results confirmed. Endline Project data were entered directly into Epi Info 7 from the completed questionnaires by trained Curamericas/Guatemala data technicians who performed cross-checking of all data entries. Epi Info 7.1 was then used to obtain lists, frequencies, and tables that included calculated percentages, means, medians, and ranges for all indicators and demographic data points, as well as confidence intervals/margins of error for each proportional result. Statistical significance was determined by obtaining *p*-values and comparing differences for the same indicator for the baseline and final KPC survey in each Area using Epi Info Stat Calc. All KPC survey data are available online [25, 26]. A standard difference-in-differences analysis was carried out by computing the difference from baseline to endline in Area A for a given indicator and then comparing it to the difference from baseline to endline for the same indicator in Area B [27]. The statistical significance for the difference-in-differences analysis was assessed using a z-test based on the variances of its four component proportions [28].

## Results

### Socio-demographic characteristics of respondents

There were almost no statistically significant differences between Area A and Area B in the socioeconomic characteristics of the households surveyed at baseline and at endline (level of education, mean age, marital status, employment and Spanish speaking ability) [25, 26]. The two Areas are quite comparable and underwent no notable changes over the period of Project implementation. Therefore the two Areas were and remained demographically similar and comparable. The median level of education among mothers of children 0- < 24 months of age was only 3 years; 98% preferred to speak their native Maya language (Chuj, Akateko, or Q’anjob’al); and fewer than half were able to communicate in Spanish. The vast majority were married and were housewives without outside employment.

Table 2 contains the complete set of findings regarding changes in intervention indicators. Baseline and endline findings are presented for each Area with their associated 95% confidence intervals. *P*-values are presented for: (1) the difference in the baseline and endline values for each indicator by Area and (2) the difference-in-differences values for each indicator by Area.

**Table 2:** Outcomes for 34 indicators comparing baseline knowledge, practice and coverage (KPC) results with endline results in Areas A and B of the Curamericas/Guatemala Maternal and Child Health Project, 2011–2015, with difference-in-differences analysis of changes from baseline to endline to test the hypothesis of whether the improvements in coverage were greater in Area A than in Area B

Outcome Indicator	Area A			Area B			Difference in differences (Area A difference minus Area B difference)	***p***-value for difference in differences	Hypothesis supported **
	Baseline percentage (numerator/denominator) (95% CI)	Endline percentage (numerator/denominator) (95% CI)	Percentage difference A (endline minus baseline) (95% CI) ***p*** value	Baseline percentage (numerator/denominator) (95%CI)	Endline percentage (numerator/denominator) (95% CI)	Percentage difference B (endline minus baseline) (95% CI) ***p*** value			
**Antenatal care**									
*At least 4 quality antenatal care checks by a skilled provider (doctor, nurse, nurse-midwife) during most recent pregnancy	13.4% (40/299) (9.5, 17.3%)	65.0% (195/300) (59.5, 70.5%)	51.6% (44.8, 58.4%) < 0.001	6.3% (19/300) (3.5, 9.1%)	53.3% (160/300) (47.6, 59.1%)	47.0% (40.6, 53.4%) < 0.001	+ 4.6%	0.321	No
*Tetanus toxoid immunization during most recent pregnancy	63.2% (189/299) (57.6, 68.8%)	67.7% (203/300) (62.3, 73.1%)	4.5% (−3.3, 12.2%) 0.25	63.0% (189/300) (55.7, 67.0%)	62.3% (187/300) (56.7, 67.9%)	−.07% (−6.9, 8.9%) 0.80	+ 5.2%	0.355	No
*Iron/folate for at least 90 days during most recent pregnancy	21.7% (65/299) (17.0, 26.5%)	64.3% (193/300) (58.8, 69.9%)	42.6% (35.3, 49.9%) < 0.001	10.0% (30/300) (6.5, 13.5%)	26.3% (79/300) (21.2, 31.4%)	16.3% (10,2, 22.5%) < 0.001	+ 26.3%	< 0.001	Yes
Knowledge of at least 2 danger signs during pregnancy	22.1% (66/299) (17.3, 26.9%)	78.3% (235/300) (73.6, 83.1%)	56.2% (49.5, 63.0%) < 0.001	21.3% (64/300) (16.6, 26.1%)	66.3% (199/300) (60.9, 71.8%)	45.0% (37.8%, 52..2%) < 0.001	+ 11.2%	0.023	Yes
**Delivery care**									
Knowledge of at least 2 danger signs during delivery	13.4% (40/299) (9.5, 17.3%)	66.3% (199/300) (60.9, 71.8%)	53.0% (46.2, 59.7%) < 0.001	13.3% (40/300) (9.4, 17.3%)	53.7% (161/300) (47.9, 59.4%)	40.3% (33.4, 47.3%) < 0.001	+ 12.5%	0.009	Yes
Mothers of children 0- < 24 months of age who reported that their community had in place an emergency response transport plan^a^	29.4% (88/299) (24.2, 34.7%)	44.7% (134/300) (38.9, 50.4%)	15.2% (7.4, 23.0%) < 0.001	37.0% (111/300) (31.4, 42.6%)	52.7% (158/300) (46.9, 58.4%)	15.7% (7.6 23.7%) < 0.001	−0.4%	1.0	No
Children 0- < 24 months of age whose births were attended by a skilled health worker (doctor, nurse, nurse-midwife)	15.4% (46/299) (11.2, 19.6%)	29.3% (88/300) (24.1, 34.6%)	14.0% (7.2, 20.7%) < 0.001	6.0% (18/300) (3.3, 8.7%)	13.7% (41/300) (9.7, 17.6%)	7.7% (2.8, 12.5%) 0.001	+ 6.2%	0.12	No
Births among children 0- < 24 months of age attended by a *comadrona* at home	77.6% 232/299 (72.8, 82.4%)	71.3% 214/300 (66.1, 76.6%)	−6.3% (−13.4, 0.8%) 0.078	85.3% 256/300 (81.2, 89.4%)	71.3% 214/300 (66.1, 76.6%)	−14.0% (−20.6, −7.4%) < 0.001	+ 7.7%	0.11	No
Births that took place in a health facility (hospital, clinic, or Birthing Center)	16.4% (49/299) (12.1, 20.7%)	28.7% (86/300) (23.4, 33.9%)	12.3% (5.5, 19.0%) < 0.001	6.7% (20/300) (3.8%, 9,5%)	13.0% (39/300) (9.1, 16.9%)	6.3% (1.5, 11.2%) 0.009	+ 6.0%	0.152	No
Births receiving active management of third stage of labor (AMTSL) during the most recent delivery^b^	9.4% (28/299) (6.0, 12.7%)	20.0% (60/300) (15.4, 24.6%)	10.6% (4.9, 16.4%) < 0.001	7.0% (21/300) (4.0, 9.9%)	11.0% (33/300) (7.4, 14.6%)	4.0% (−0.7, 8.7%) 0.086	+ 6.6%	0.072	No
Women with a child 0- < 24 months of age whose previous delivery was by cesarean section	N/A	8.7% (26/300) (5.3, 12.1%)	N/A	N/A	2.3% (7/300) (0.4, 4.2%)	N/A	N/A	N/A	N/A
**Postpartum care**									
Knowledge of at least 2 postpartum danger signs	17.1% (51/299) (12.7, 21.4%)	66.3% (199/300) (60.9, 71.8%)	49.3% (42.3, 56.3%) < 0.001	18.7% (56/300) (14.2, 23.2%)	54.3% (163/300) (48.6, 60.1%)	35.7% (28.4, 43.0%) < 0.001	+ 13.6%	0.007	Yes
Postpartum visit for the mother and newborn within 48 hours after delivery	22.4% (67/299) (17.6, 27.2%)	39.0% (117/300) (33.4, 44.6%)	16.6% (9.2, 24.0%) < 0.001	16.0% (48/300) (11.8, 20.2%)	18.3% (55/300) (13.9, 22.8%)	2.3% (−3.8, 8.5%) 0.448	+ 14.3%	0.003	Yes
*Percentage of mothers of children 0- < 24 months who received vitamin A supplementation within 2 months postpartum (card verified or maternal recall)	22.1% (66/299) (17.3, 26.9%)	47.7% (143/300) (41.9, 54.4%)	25.6% (18.1, 33.1%) < 0.001	17.0% (51/300) (12.7, 21.3%)	26.7% (80/300) (21.6, 31.8%)	9.7% (3.0, 16.4%) 0.004	+ 15.9%	0.002	Yes
**Newborn care**									
Knowledge of at least 2 neonatal danger signs	27.4% (82/299) (22.3, 32.6%)	64.7% (194/300) (59.1, 70.2%)	37.3% (29.7, 44.8%) < 0.001	29.7% (89/300) (24.4, 34.9%)	58.7% (176/300) (53.0, 64.4%)	29.0% (21.2, 36.8%) < 0.001	+ 8.3%	0.128	No
Essential Newborn Care during the most recent delivery^c^	6.0% (18/299) (3.3, 8.8%)	39.0% (117/300) (33.4, 44.6%)	33.0% (26.7, 39.2%) < 0.001	5.0% (15/300) (2.5, 7.5%)	31.0% (93/300) (25.7, 36.3%)	26.0% (20;1, 31.9%) < 0.001	+ 7.0%	0.105	No
**Birth spacing and family planning**									
Knowledge of at least 2 risks associated with pregnancy intervals < 24 months	6.4% (19/299) (3.5, 9.2%)	46.7% (140/300) (40.9, 52.4%)	40.3% (33.9, 46.7%) < 0.001	12.0% (36/300) (8.2, 15.8%)	33.7% (101/300) (28.2, 39.1%)	21.7% (15.0, 28.3%) < 0.001	+ 18.6%	< 0.001	Yes
*Current modern contraceptive use among non-pregnant women	40.0% (107/267) (34.2, 46.0%)	40.0% (102/255) (33.9, 46.3%)	0.0% (−8.7, 8.9%) 1.000	32.0% (81/265) (25.1, 36.5%)	29.6% (75/253) (24.1, 35.7%)	− 2.4% (−7.4, 9.2%) 0.848	+ 2.4%	0.886	No
*Birth interval < 24 m between most recent 2 deliveries	32.7% (75/299) (26.9, 39.0%)	24.7% (56/226) (19.2, 30.9%)	− 8.0% (− 28.1, 16.3%) 0.097	33.5% (77/230) (27.6, 38.7%)	35.9% (75/209) (29.6, 42.5%)	2.4% (−11.4, 7.9%) 0.392	−5.7%	1.000	No
**Child nutrition**									
Exclusive breastfeeding (children 0- < 6 months of age) in previous 24 hours	75.0% (63/84) (65.5, 84.4%)	82.0% (73/89) (73.9, 90.2%)	7.0% (5.4, 19.5%) 0.260	79.2% (57/72) (69.6, 88.7%)	71.6% (58/81) (61.6, 81.6%)	− 7.6% (− 21.4, 6.3%) 0.275	+ 14.6%	0.002	Yes
Proper infant and young child feeding (children 6- < 24 months of age)	53.0% (114/215) (46.2, 59.8%)	74.3% (156,210) (68.3, 80.3%)	21.3% (12.2, 30.4%) < 0.001	56.1% (128/228) (49.6, 62.7%)	65.3% (143/219) (58.9%, 71.7%	9.2% (0.0, 18.4%) 0.046	+ 12.1%	0.028	Yes
*Vitamin A supplementation for child 6- < 24 months in last 6 months	79.1% (170/215) (73.5, 84.6%)	74.3% (156/210) (68.3, 80.3%)	−4.8% (−13.0, 3.4%) 0.243	73.7% (168/228) (67.9, 79.5%)	67.1% (147/219) (69.8, 73.5%)	− 6.6% (−15.2, 2.1%) 0.128	+ 1.8%	0.732	No
**Childhood pneumonia**									
Appropriate care seeking for child with symptoms of pneumonia	26.1% (20/77) (16.0, 36.0%)	51.6% (32/62) (38.9, 64.3%)	25.6% (9.5, 41.8%) 0.002	20.5% (16/78) (11.4, 29.7%)	46.6% (27/58) (33.5, 59.7%)	26.0% (10.1, 42.0%) 0.001	−0.5%	1.000	No
**Childhood diarrhea**									
Use of oral rehydration therapy (or a recommended home fluid) during the most recent diarrheal episode	28.3% (34/120) (20.1, 36.6%)	40.8% (42/103) (31.1, 50.5%)	12.4% (−0.3, 25.2%) 0.05	30.5% (36/118) (22.0, 39.0%)	40.2% (47/117) (31.1, 49.2%)	9.7% (−2.7%), 22.1%) 0.119	+ 2.8%	0.638	No
Increased fluid intake during the most recent diarrheal episode	7.5% (9/120) (2.7%, 12.3%)	18.5% (19/103) (10.8, 26.1%)	11.0% (1.9, 20.0%) 0.015	7.6% (9/118) (2.7%, 12.5%),	16.2% (19/117) (9.4%, 23.1%)	8.6% (0.2, 17.0%) 0.040	+ 2.3%	0.540	No
Same/increased food intake during the most recent diarrheal episode	0.0% (0/120) (0.0, 2.5%)	0.0% (0/103) (0.0, 2.9%)	0.0% (0.0, 0.0%) N/A	2.5% (3/118) (0.0, 5.4%)	5.1% (6/117) (1.0, 9.2%)	2.6% (−2.4, 7.6%) 0.301	−2.6%	1.000	No
*Zinc treatment for the most recent diarrheal episode	6.7% (8/120) (2.1, 11.2%)	10.7% (11/103) (4.6, 16.8%)	4.0% (−3.6, 1.6%) 0.291	1.7% (2/118) (0.0, 4.1%)	10.3% (12/117) (4.6%, 15/9%)	8.6% (2.5, 14.7%) 0.005	−4.6%	1.000	No
**Childhood immunization**									
*Measles Immunization children 12- < 24 months	79.3% (96/121) (72.0, 86.7%)	64.8% (79/122) (56.1, 73.4%)	−14.5% (−25.9, −3.2%) 0.010	78.9% (116/147) (72.2, 85.6%)	55.5% (66/119) (46.4, 64.6%)	− 23.5% (− 34.8, −12.1%) < 0.001	+ 8.9%	0.092	No
*Complete vaccination coverage children 12- < 24 months (BCG, PENTA 1–3, polio 1–3, measles)	73.6% (89/121) (65.5, 81.6%)	56.6% (69/122) (47.6, 65.5%)	−17.0% (− 29.0, −5.0%) 0.005	68.7% (101/147) (61.1, 76.4%)	50.4% (60/119) (41.3, 59.6%)	−18.3% (− 30.2, −6.3%) 0.002	+ 1.3%	0.796	No
**WASH (wáter, sanitation and hygiene)**									
Regular point-of-use water treatment	66.6% (199/299) (61.1, 72.0%)	97.7% (293/300) (95.9, 99.4%)	31.1% (25.4, 36.8%) < 0.001	58.3% (175/300) (52.6, 64.0%)	97.7% (293/300) (95.9, 99.4%)	39.4% (33.4, 45.3%) < 0.001	−8.3%	1.000	No
Safe water storage	11.7% (35/299) (8.0, 15.4%)	28.0% (84/300) (22.8, 33.2%)	16.3% (9.9, 22.7%) < 0.001	10.3% (31/300) (6.8, 13.8%)	26.0% (78/300) (20.9, 31.1%)	15.7% (9.5, 21.8%) < 0.001	+ 0.6%	0.888	No
Safe disposal of child’s feces the last time s/he defecated	43.1% (129/299) (37.4, 48.9%)	45.0% (135/300) (39.3, 50.7%)	1.9% (−6.3, 10.0% 0.647	38.7% (116/300) (33.0, 44.3%)	52.0% (156/300) (46.2, 57.8%)	13.3% (5.3, 21.4%) < 0.001	−11.4%	1.000	No
Appropriate hand washing station in home (with water, soap, and soap recipient)	2.3% (7/299) (0.6, 4.1%)	44.7% (134/300) (38.9, 50.4%)	42.3% (36.3, 48.3%) < 0.001	2.3% (7/300) (0.6, 4.1%)	44.0% (132/300) (38.3, 49.7%)	41.7% (35.7, 47.7%) < 0.001	+ 0.7%	0.877	No
Knowledge of the 4 critical times when hands should be washing (after defecating, before preparing food, after cleaning a child who has defecated, before feeding a child)	1.3% (4/299) (0.0, 2.7%)	34.0% (102/300) (28.5, 39.5%)	32.7% (27.0, 38.3%) < 0.001	1.7% (5/300) (0.2, 3.1%)	28.7% (86/300) (23.4, 33.9%)	27.0% (21.6, 32.4%) (< 0.001)	−5.7%	0.148	No

*NA* not assessed, *CI* confidence interval

*Denotes indicators (n=10) that were tracked by the Project but depended on services delivered through the government-run PEC Program and therefore considered outside of the Project’s control

**The difference in differences is positive and statistically significant

^a^A plan that would provide transport for the mother and/or her newborn to the nearest health facility in the event of (1) a difficult delivery or (2) danger signs in pregnancy or during the postpartum period

^b^AMTSL: active management of the third stage of labor (controlled cord traction, uterine massage, and administration of a uterotonic drug)

^c^Essential Newborn Care: clean umbilical cord care, immediate breastfeeding, thermal care

### Maternal and newborn care

The final evaluation results reveal marked, statistically significant increases from baseline to endline in both Areas in nearly all indicators related to maternal and newborn care, as shown in Table 2.

#### Antenatal care

Three of the four antenatal care indicators showed marked, statistically significant increases in coverage from baseline to endline in both Areas A and B: quality antenatal care, iron/folate supplementation, and knowledge of prenatal danger signs. Only tetanus immunizations showed no significant change.

The percentage of mothers who reported receiving four quality antenatal care checks from a qualified health professional during their most recent pregnancy increased 4.9-fold in Area A and 8.5-fold in Area B. Iron supplementation for at least 90 days during the most recent pregnancy increased 3.0-fold in Area A and 2.6-fold in Area B. Knowledge of pregnancy-related danger signs increased 3.5-fold in Area A and 3.1-fold in Area B. However, mothers reporting two tetanus toxoid vaccinations prior to their most recent childbirth did not show any statistically significant improvements from baseline to endline in either Area.

Endline coverage of quality antenatal care, iron supplementation, and knowledge of danger signs in pregnancy were significantly higher in Area A than in Area B. No significant difference between Areas was noted for the endline tetanus toxoid immunization coverage, holding steady at 62–68% in both Areas at baseline and endline.

#### Delivery care

Marked statistically significant improvements from baseline to endline were observed for all but two of the seven delivery-related indicators in both Areas A and B. The only indicators without a statistically significant improvement were: (1) percentage of births among children 0- < 24 months of age delivered at home by a *comadrona* in Area A, which fell from 77.6% at baseline to 71.3% at endline, and (2) active management of the third stage of labor (AMTSL) in Area B, which saw a modest increase from 7.0 to 11.0%. Both indicators just barely missed statistical significance (*p* = 0.078 and *p* = 0.086, respectively). Knowledge of danger signs during delivery rose 4.9-fold in Area A and 4.0-fold in Area B. The percentage of mothers who reported that their communities had an emergency response transport plan increased in both Areas A and B: by 15 percentage points in Area A and 16 percentage points in Area B.

The percentage of deliveries attended by a skilled professional birth attendant (doctor, registered nurse, or nurse-midwife) doubled in both Areas (a 1.9-fold increase in Area A and a 2.3-fold increase in Area B), although the endline percentages were still modest (29.3% in Area A and 13.7% in Area B). The percentage of births attended by a *comadrona* declined modestly in both areas: by 6 percentage points in Area A and by 14 percentage points in Area B. In Area B, the difference was statistically significant. The percentage of deliveries taking place in a facility (including a Community Birthing Center) doubled in both Areas (from 16.4 to 28.7% in Area A and from 6.7 to 13.0% in Area B). Information about cesarean section was not collected at baseline, but at endline 8.7% of births in Area A were by cesarean section. The frequency of cesarean section in Area B at endline was low at 2.3%.

#### Postpartum care

Knowledge of at least two danger signs during the postpartum period rose 3.9-fold in Area A and 2.9-fold in Area B, and the percentage of women who reported a postpartum visit for themselves and their newborn within 48 hours of delivery rose 1.7-fold in Area A but did not increase significantly in Area B.^2^ These two postpartum indicators had a higher value at endline in Area A than the respective indicator in Area B. Vitamin supplementation for mothers within 2 months postpartum increased 2.2 fold in Area A and 1.6 fold in Area B, both statistically significant increases.

#### Newborn care

Maternal knowledge of at least two danger signs during the neonatal period rose 2.4-fold in Area A and 2.0-fold in Area B. The percentage of newborns receiving Essential Newborn Care (clean umbilical cord care, thermal protection, and immediate breastfeeding) increased 6.5-fold in Area A and 6.2-fold in Area B. These improvements were statistically significant in both areas for both indicators.

#### Birth spacing and family planning

The percentage of mothers of children 0- < 24 months of age who knew at least two risks of having a birth interval of less than 24 months increased substantially and significantly from baseline to endline in both Area; however, despite this increase in knowledge, the percentage of non-pregnant women who reported using a modern contraceptive method was effectively unchanged from baseline to endline in both Areas. Nonetheless, the percentage of women whose interval between the births of her two youngest children was equal to or less than 24 months declined in Area A. This decline approached the threshold for statistical significance (p=0.097. In Area B there was no decline.

#### Child nutrition

Though considerable Project effort was dedicated to this topic, indicators for child feeding showed minimal changes during the period of evaluation. The prevalence of exclusive breastfeeding did not change in either Area A or Area B, though fortunately the baseline prevalence was reasonably high (75–79%). Proper feeding from 6 to < 24 months of age showed some improvements (by 21 percentage points in Area A and by 9 percentage points in Area B, both statistically significant). Childhood vitamin A supplementation did not improve in either area. The following paper in this supplement [16] explores changes in childhood nutritional status.

#### Childhood pneumonia

The percentage of children with cough and rapid/difficult breathing in the previous 2 weeks ranged between 19.3 and 26.0% based on the baseline and endline surveys in Areas A and B. Prompt care seeking and treatment from a health worker for a child with symptoms of pneumonia increased significantly in both Areas from baseline to endline (a 2.0-fold increase in Area A and a 2.3-fold increase in Area B).

#### Childhood diarrhea

The percentage of mothers who reported that their child had a diarrheal episode in the 2 weeks before the interview varied between 34.3 and 40.1% based on the baseline and endline surveys in Areas A and B. The percentage of mothers who provided these children with oral rehydration solution or recommended home fluids improved significantly by 12 percentage points in Area A, and it increased by 10 percentage points in Area B, but this change was not statistically significant. Statistically significant increases from very low baseline levels are seen in both Areas in the percentage of children with diarrhea who were offered increased fluid intake. The use of zinc to shorten and ameliorate diarrhea episodes increased in both Areas, but the increase was statistically significant only in Area B. No significant changes were seen in offering the same/increased food intake for children with diarrhea, which remained quite low.

#### Childhood immunization

Both childhood immunization indicators – measles immunization coverage and full immunization coverage (BCG, pentavalent [PENTA] and polio) for children 12- < 24 months of age – *decreased* significantly from baseline to endline in both Areas (a decline of 15 and 23 percentage points in Areas A and B, respectively, for measles immunization and a decline of 17 and 18 percentage points in Areas A and B respectively for the complete immunization series in the two Areas). We discuss possible reasons for this below.

#### WASH (water, sanitation, and hygiene)

The endline KPC survey documented outstanding results and significant improvements over baseline levels in nearly every WASH indicator. The percentage of mothers reporting appropriate point-of-use water treatment and the percentage of mothers reporting safe water storage practices increased significantly in both Areas A and B. The percentage of mothers who reported that their household had an appropriate hand washing station (with soap, water, and water container) and the percentage of mothers who reported washing their hands at all four critical moments (after defecating, after cleaning a child who had defecated, before preparing food, and before feeding a child) both increased substantially and significantly in both Areas. Safe disposal of a child’s feces (in a latrine or toilet connected to a septic system) the last time s/he defecated was essentially unchanged in Area A but increased significantly in Area B.

#### Comparison of project outcomes in Area A with Area B

One of our original hypotheses was that the outcomes in Area A would be more favorable than in Area B because of the longer implementation time in Area A (41 months) compared to Area B (20 months). This hypothesis was partially affirmed.

A difference-in-difference analysis was carried out that assessed the degree to which the difference in coverage (comparing endline with baseline) was greater in Area A than in Area B. This analysis tests the hypothesis that the changes in coverage of Project indicators were greater in Area A than in Area B because the duration of the interventions was almost twice as long in Area A as in Area B, thereby giving strength to the argument of causality that the interventions were responsible for the observed changes in coverage rather than some other non-measured influence. If the difference in differences is positive (i.e., a difference between endline and baseline for Area A is greater than in Area B) and the difference is statistically significant, then the hypothesis is supported. As shown in Table 2 (in the far right ot the table), the hypothesis was supported for 9 of the 33 of the indicators included in the analysis. In two cases, the level of statistical significance was nearly reached (*p* = 0.072 and *p* = 0.092). There were no cases in which the difference in differences was negative and statistically significant. The difference in differences was ≥ + 5% for 17 of the 33 indicators.

#### Summary of findings from the perspective of type of intervention and level of indicator improvement

Table 3 provides a summary of the strength of effect of the Project on the 24 maternal and child health indicators that were under the Project’s control (and were not affected by the termination of the PEC Program) and that are now grouped into three new categories: knowledge indicators, household practices, and health facility utilization/treatment. Nine indicators that were affected by the PEC Program shutdown, were deleted from the analysis as was one other indicator (delivery at home by a *comadrona*) since it is the obverse of the indicators for delivery in a health facility by a skilled attendant. In addition, the indicator regarding cesarean section is not included since no baseline data are available.

**Table 3:**
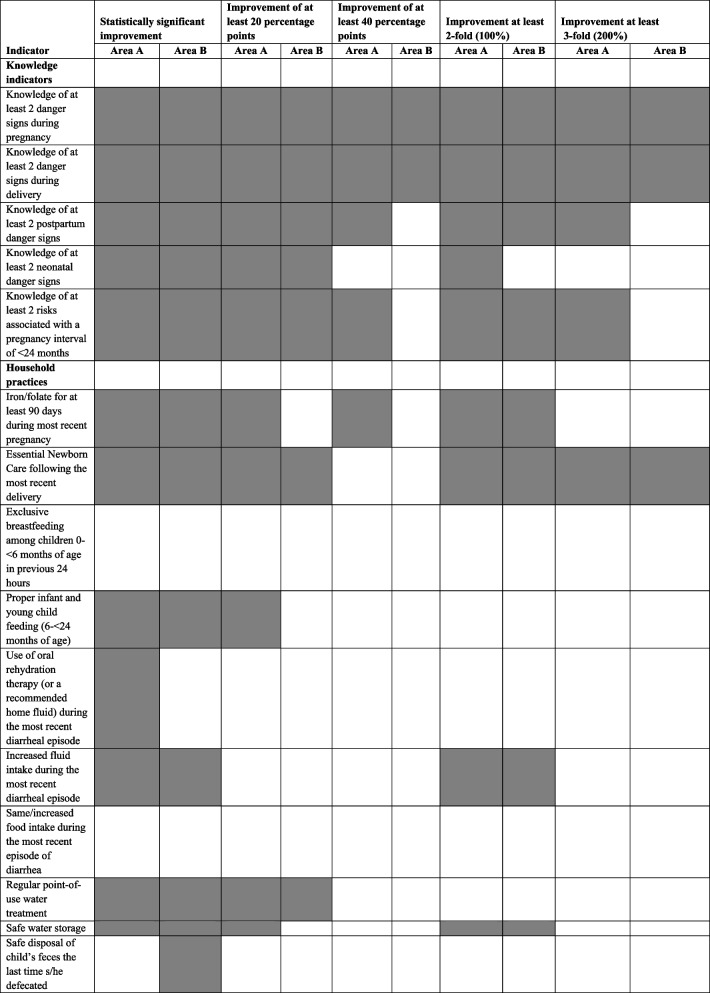

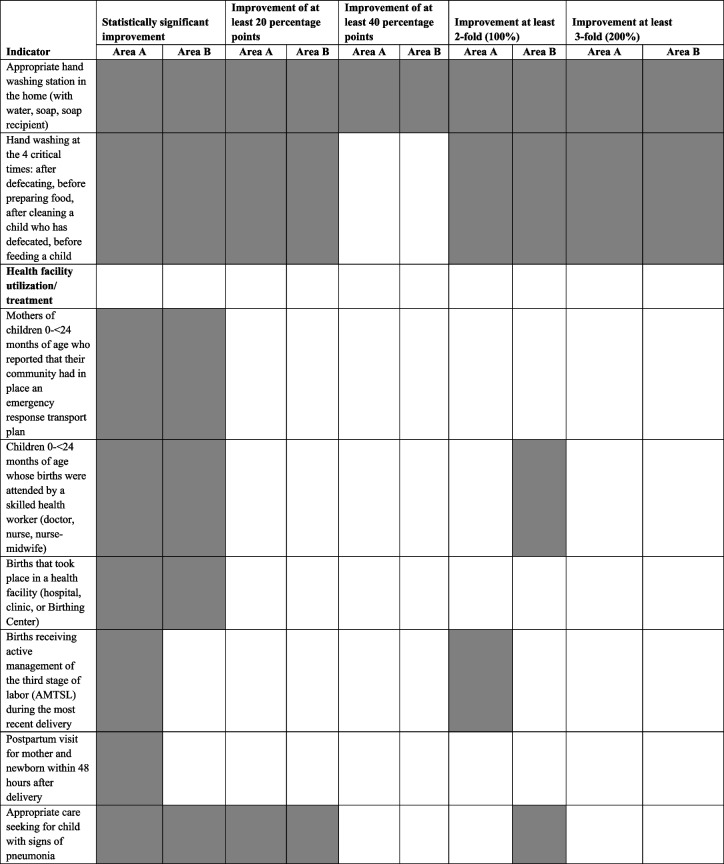
Summary of strength of effect of the Curamericas/Guatemala Maternal and Child Health Project, 2011–2015, for 25 maternal and child health indicators for activities under the Project’s control, organized by knowledge indicators, household practices, and health facility utilization/treatment^a^

^a^The following indicators were for activities under the purview of the PEC Program and are not included in this analysis: antenatal care, vitamin A administration to mothers, vitamin A administration to children, maternal tetanus immunization, use of contraception, birth interval less than 24 months, zinc treatment for childhood diarrhea, child measles immunization, and complete child immunization. Another indicator was also not included: delivery at home by a *comadrona* since it is essentially the obverse of delivery in a facility by a skilled attendant. In addition, the indicator for cesarean section was not included since there was not baseline measurement

We have indicated in Table 3 whether there was a statistically significant improvement in coverage, an improvement of at least 20 percentage points, an improvement of at least 40 percentage points, a 2-fold (100%) improvement, or a 3-fold (200%) improvement. In Area A, the improvements were statistically significant for 20 of the 24 indicators, and in Area B, the improvements were statistically significant for 18 of the 24 indicators. For 14 of 24 indicators in Area A and for 11 of the 24 indicators in Area B there was at least a 20-percentage-point increase. For 7 of 24 indicators in Area A and for 3 of 24 indicators in Area B, there was a 40-percentage-point increase. The increases represented a 2-fold (100%) increase for 12 of the 24 indicators in Area A and for 12 of the 24 indicators in Area B. Finally, there was a 3-fold (200%) increase in 7 of the 24 indicators in Area A and in 5 of the 24 indicators in Area B.

Of note is the marked improvements in all the knowledge indicators. All the levels of indicator improvement were achieved for 4 of the 5 knowledge indicators in Area A and for 3 of the 5 in Area B. Among the household practice indicators, the strongest progress was made for handwashing – both for the presence of a handwashing station in the home and for appropriate handwashing practices.

### Comparison of endline levels of coverage indicators with levels for broader population groups in Guatemala

In addition to assessing whether coverage indicators increased from baseline to endline and whether the increases were greater in Area A than in Area B, our original hypotheses and research questions outlined in Paper 2 of this series [15] included whether coverage levels at endline were more favorable than in surrounding municipalities that were adjacent to the Project Area but outside of it and also whether the levels were more favorable than in the rural population of the Department of Huehuetenango. We were not able to locate data to test the hypothesis for adjacent municipalities nor for the rural population of the Department of Huehuetenango. However, we were able to locate data for a number of the indicators in our study for the entire Department of Huehuetenango, which includes both urban and rural areas. The findings, which are reported in the 2014-15 National Demographic and Health Survey [29] are shown in Table 4.

**Table 4:** Comparison of endline levels of coverage indicators for Curamericas/Guatemala Maternal and Child Health Project with levels for broader population groups in Guatemala

Indicator	Project endline levels, 2015		Levels for the Department of Huehuetenango (reported in 2014-15 DHS survey [29])	Is the indicator level in Project Area more favorable than in the Department of Huehuetenango?
	Area A	Area B		
**Antenatal care**				
Tetanus toxoid immunization during most recent pregnancy	67.7	62.3	66.7	Yes (Area A only and barely)
Iron/folate for at least 90 days during most recent pregnancy	64.3	26.3	19.8	Yes
**Delivery care**				
Children 0- < 24 months of age whose births were attended by a skilled health worker (doctor, nurse, nurse-midwife)	29.3	13.7	39.1	No
Births among children 0- < 24 months of age attended by a *comadrona* at home	71.3	71.3	60.6	No^a^
Births that took place in a health facility (hospital, clinic, or Birthing Center)	28.7	13.0	39.0	No^b^
Women with a child 0- < 24 months of age whose previous delivery was by cesarean section	8.7	2.3	14.6	No
**Postpartum care**				
Postpartum visit for the mother and newborn within 48 hours after delivery	39.0	18.3	73.4	No
Percentage of mothers of children 0- < 24 months who received vitamin A supplementation within 2 months postpartum	47.7	26.7	26.2	Yes (Area A only)
**Newborn care**				
No indicators available for comparison				
**Birth spacing and family planning**				
Current use of modern contraception	34.0	25.0	40.1	No
**Child feeding**				
Exclusive breastfeeding among children 0- < 6 months of age in previous 24 hours	82.0	71.6	71.1	Yes (Area A only)
Vitamin A supplementation for child 6- < 24 months of age in the previous 6 months	74.3	67.1	44.3	Yes
**Childhood pneumonia**				
Appropriate care seeking for child with symptoms of pneumonia	51.6	46.6	47.5	Yes (barely, in Area A only)
**Childhood diarrhea**				
Use of oral rehydration therapy (or a recommended home fluid) during diarrheal episode	40.8	40.2	63.4	No
Increased fluid intake during diarrheal episode	11.0	16.2	23.6	No
Same/increased food intake during diarrheal episode	0.0	2.6	1.7	Yes (barely, in Area B only)
**Childhood immunizations**				
Measles immunization in children 12- < 24 months of age	64.8	55.5	46.5	Yes
Complete vaccination coverage (BCG, PENTA 1–3, polio 1–3, measles) among children 12- < 24 months of age	56.6	68.7	43.7	Yes
**WASH (water, sanitation and hygiene)**				
No indicators available for comparison				

^a^This is listed as no because the desired outcome is NOT a home birth attended by a *comadrona*

^b^ The goal for cesarean sections according to the World Health Organization is 10–15% of all deliveries. In this case, the levels in the Project Area are less favorable than in the Department of Huehuetenango

There were 17 indicators for which data from the Department of Huehuetenango were available. Of these 17, the endline results in the Project Area (either Area A or Area B or both) were more favorable than for the entire Department of Huehuetenango for the following nine indicators: (1) maternal tetanus toxoid immunization, (2) iron and folate intake during pregnancy, (3) vitamin A supplementation for postpartum mothers, (4) exclusive breastfeeding during the first 6 months of life, (5) vitamin A supplementation for children 6- < 24 months of age, (6) appropriate care seeking for a child with symptoms of pneumonia, (7) same/increased food intake during diarrhea episode, (8) measles immunization for children, and (9) complete immunization for children. (For three indicators, however, the more favorable responses were in only one of the two Areas and the difference was minimal.) It would have been much more appropriate to compare coverage levels with adjacent municipalities with similar geographical and socio-economic features and with the rural population of the Department of Huehuetenango, but these data are not available.

For the nine comparisons shown in Table 4 that support the hypothesis of a more favorable level of coverage in the Project Area, five are directly related to interventions provided by the PEC Program [vitamin A distribution to (1) mothers and (2) children, (3) maternal tetanus immunization, (4) measles immunization, and (5) full childhood immunization]. This is in spite of the fact that for the two immunization indicators, the levels for the Project Areas A and B at endline levels had actually declined from the baseline levels. Despite the limitations of the data from the comparison area, the hypothesis was partially supported.

The finding that only nine of the 17 indicators supported the hypothesis should be interpreted with the understanding that the Department of Huehuetenango (both urban and rural combined) is a highly imperfect comparison area since access to health services and general level of socioeconomic development is more favorable than in the Project Area. In spite of impressive improvements of many indicators in the Project Area, the endline levels still remained lower than for the Department of Huehuetenango as a whole. One example is the percentage of births that took place in a health facility. Even though the levels doubled from baseline to endline in both Areas, the endline levels (28.7 and 13.0%) were far below the level for the entire Department (39.0%).

## Discussion

The Project achieved statistically significant improvements in the coverage of key maternal and child health indicators in isolated rural mountainous communities of the Department of Huehuetenango. These improvements included marked increases in the population coverage of knowledge of important information about maternal and child health, marked improvements in household behaviors that promote maternal and child health, and marked improvements in health care utilization of key maternal and child health services. As far as we know, this is the most extensive and complete assessment of changes in population coverage of evidence-based maternal and child health interventions for a specific project that has been published in the peer-reviewed literature. Furthermore, the improvements in population coverage of evidence-based interventions documented here over such a short period of time are among the most notable reported as well.

The results reflect a remarkable impact of the Project’s CBIO+ approach on key knowledge, practices, and health care utilization that are known to be important for improving maternal and child health. Appreciating the substantive significance of each of these improvements depends on the specific indicator and on its baseline and endline levels. We have tried to assess the substantive significance of these improvements for the indicators over which the Project had control by indicating, not only whether the improvement was statistically significant, but also the magnitude of the increase in terms of percentage points and in terms of percentage increase from the baseline level. For 20 of these 24 indicators in Area A and for 18 of these 24 indicators in Area B, the results were favorable and statistically significant. We assessed levels of indicator improvement calculating if the indicator coverage increased by at least 20 percentage points, at least 40 percentage points, at least two-fold, or at least three-fold. If the improvement was three-fold and statistically significant, we consider the level of indicator improvement for the intervention to be extremely high.

For 3 of these 24 indicators, high levels of indicator improvement were achieved for both Areas for (1) knowledge of dangers signs during pregnancy, (2) knowledge of danger signs during delivery, and (3) presence of a handwashing station in the home. The specific indicator that showed the greatest improvement was handwashing (with a 26.1- and 16.9-fold increase in Areas A and B respectively). Iron and folate use during pregnancy, knowledge of danger signs during pregnancy, and knowledge of danger signs during delivery also demonstrated remarkable improvements.

The difference-in-differences analysis comparing Area A and Area B indicates that more favorable outcomes were achieved. This finding supports three important conclusions: (1) the findings are consistent with a dose-response effect, providing supporting evidence that the Project itself was responsible for the changes in intervention coverage; (2) if the Project activities in Area B had been implemented for a longer period of time, the results in Area B would probably have been even more notable; and (3) even though the outcomes in Area B were not quite as favorable as those in Area A, the improvements in Area B were nonetheless quite noteworthy given the short period of intervention implementation – 20 months.  Thus, the quickness with which the “full-dose effect” can be achieved through CBIO+ is particularly notable.

It is unfortunate that we did not measure the incidence of cesarean section at baseline. However, the incidences observed at endline are instructive: 8.7% in Area A and 2.3% in Area B. This is a difference that is highly significant statistically (*p* < 0.001). The current guidance from the World Health Organization is based on the finding that maternal and neonatal mortality rates improve as the cesarean rate approaches 10% and therefore considers the ideal rate to be between 10 and 15% [30]. Thus, we can conclude that Area A met this standard but not Area B. It would be interesting to know what the baseline rates were and whether they improved in each Area between baseline and endline.

There was a strong sense of local ownership of the assessment process. Curamericas/Guatemala staff gained experience in the evaluation process through their participation in both the baseline and endline surveys as interview supervisors and data technicians, as well as by providing input on the drafting of the KPC questionnaires. The inclusion of municipal, community, and MSPAS officials participating in the data collection in remote communities and households was particularly valuable since the findings and results were of great interest to all stakeholders.

Two indicators show no improvement: exclusive breastfeeding and same/increased food intake during diarrhea. The baseline for exclusive breastfeeding was already high (75–79%), so further improvements would have been challenging. The lack of progress on same/increased food intake during episodes of diarrhea reflect a strongly held cultural value that proved to be resistant to change, namely that giving food during episodes of childhood diarrhea would exacerbate the diarrhea.

Although the gains in coverage of key indicators were generally greater in Area A than in Area B, the progress made in Area B after only 20 months of implementation is noteworthy. Statistically significant gains in coverage were achieved in Area B for all but 6 of the 24 indicators of activities for which the Project had control. There were only 3 of 21 cases in which a statistically significant increase was achieved in Area A but not achieved in Area B, and in only 1 case, a statistically significant increase was achieved in Area B but not in Area A. This indicates that the CBIO+ approach can achieve notable increases in coverage quite quickly.

### Contextual explanation for lack of improvement of PEC-related indicators

Certain indicators showed no improvement and in some cases a decline. These were all related to activities under the purview of the PEC Program: use of family planning, vitamin A supplementation for children and mothers, and all immunization indicators (tetanus toxoid immunization coverage for mothers, measles immunization coverage for children, and complete immunization coverage for children). The lack of improvement in these time-sensitive indicators can be attributed to the national shutdown in 2014 of the PEC Program. Antenatal care and distribution of iron/folate were also provided by the PEC Program, but the measurement of these indicators at endline referred to activities that had taken place prior to the shutdown. The shutdown was produced by political turmoil in national politics and in the MSPAS centrally, leading to a lack of funding for the program. The closure of the PEC Program made it impossible for the Ambulatory Nurses hired for the PEC Program to continue their work and to provide family planning services, vaccinations, and vitamin A supplements. Fortunately, the Project was able to develop a partial stop-gap solution by working with the non-governmental organization Medicines for Humanity, which made it possible for pregnant women to obtain iron and folate and other essential medicines at *botiquines – *small self-sustaining drug shops based in the Birthing Center.

### Limitations

There are several limitations of our assessment that could have affected our findings. (1) The strength of Project implementation varied from community to community. Most communities were highly receptive and cooperative, but there were some that were reluctant to cooperate initially or even refused; by the end of the Project, all but two communities were full participants. The degree to which these communities were included or not in the sampling for the baseline and endline KPCs could have affected the results, but it is hard to know exactly how this might have skewed the results. (2) Although interviewers were intensely trained, many were inexperienced. This could have affected interview comprehension. (3) The results may have been affected by seasonal differences in disease incidence, with pneumonia more prevalent during the dry/cold season (December to March), when the baseline KPC was carried out and diarrhea more prevalent during the rainy season (June to October), when the endline KPC was done. (4) Oral translation of questions written in Spanish but administered in Chuj, Akateko, and Q’anjob’al could have affected comprehension and therefore could have affected the results. It is not clear how these factors (other than seasonality) might have affected our results. They could have produced a bias either toward more favorable or less favorable outcomes. Most likely, the bias could have been different for each limitation, so most likely the overall effect of these limitations was small.

Maternal recall was required for virtually all indicators except for those in which information on the maternal or child health car was extracted. This could have affected the accuracy of our findings, but it is not obvious that the findings would have been skewed to make the level of the indicator more favorable or less favorable.

There is one possible bias that could have produced more favorable results for at least a few of the indicators, and that is the so-called favorability bias: some of the respondents might have given answers that they thought the interviewer (or the Project) wanted to hear rather than what the true answer in fact was. This could have skewed findings for such indicators as safe disposal of child feces and handwashing.

Even after taking these limitations into account, we think that our findings are sufficiently robust to support our conclusion that the Project was effective in improving knowledge, practices, and care-seeking behaviors related to maternal and child health.

### Broader implications

The findings presented here support the value of the CBIO+ approach, particularly its emphasis on working directly with communities in partnership so that they become capable of improving their own health and particularly the health of mothers and children. The community partnerships developed here enabled women to become more capable of taking ownership of their own health and that of their children. Our findings are remarkable not only because of the magnitude of the increases in coverage but also because of the challenging context in which the Project was implemented. The findings are also remarkable because of the comprehensive set of population coverage indicators that demonstrated notable improvements. The CBIO+ approach used here deserves broader application in reaching isolated and marginalized populations, as we discuss further in the final paper of this supplement [22].

## Conclusion

This assessment demonstrates that the Curamericas/Guatemala Maternal and Child Health Project’s CBIO+ Approach has produced notable and substantive improvements in the population coverage of a broad range of interventions that were designed to address the epidemiological priorities for mothers and children in the rural highlands of Guatemala. The findings suggest that the CBIO+ Approach used here has potential for further development and for broader implementation, not only in Guatemala but in many other challenging low-income settings around the world.

## Data Availability

All of the Project reports, de-identified data, as well as publications about the Expanded CBIO+ Approach cited in this article are available from the corresponding author on request.

## Appendix 1

### Definition of indicators

#### Maternal and newborn care

Quality antenatal care: Percentage of mothers of children 0- < 24 months of age who had four or more antenatal visits with a skilled provider (doctor, nurse, professional midwife).

Tetanus toxoid immunization of mothers: Percentage of mothers with children 0- < 24 months of age who received at least 2 tetanus toxoid vaccinations before the birth of their youngest child.

Iron/folate supplementation during pregnancy: Percentage of mothers of children 0- < 24 months of age who took iron tablets or syrup for 90 days during their most recent pregnancy.

Knowledge of pregnancy-related danger signs: Percentage of mothers of children 0- < 24 months of age who could cite at least two pregnancy-related danger signs.

Delivery in a health facility: Percentage of children 0-<24 months of age whose births occurred in a health facility (hospital, clinic, or Birthing Center).

Community obstetric emergency response plan: Percentage of mothers of children 0- < 24 months of age who reported that their community had in place an emergency response plan that would provide transport for them and/or their newborn child to the nearest health facility in the event of danger signs during pregnancy, a difficult delivery, or danger signs during the post-partum period.

Provision of Essential Newborn Care: Percentage of children 0- < 24 months of age who received all three elements of Essential Newborn Care: thermal protection immediately after birth, clean cord care, and immediate and exclusive breastfeeding.

Active management of third stage of labor (ATMSL): Percentage of mothers of children 0- < 24 months of age who received AMTSL during their most recent delivery: a uterotonic drug; uterine massage; and controlled traction of the umbilical cord.

Knowledge of delivery-related danger signs: Percentage of mothers of children 0- < 24 months of age who could cite at least two delivery-related danger signs.

Post-partum visit for the mother and newborn: Percentage of mothers of children 0- < 24 of age who received a post-partum visit from an appropriately trained health worker within 2 days of delivery.

Knowledge of danger signs during the post-partum period: Percentage of mothers of children 0- < 24 months of age who could cite at least two danger signs during the post-partum period.

Knowledge of neonatal danger signs: Percentage of mothers of children 0- < 24 months of age who could cite at least two neonatal danger signs.

Vitamin A supplementation for mother: Percentage of mothers of children 0- < 24 months of age who received vitamin A supplementation within 2 months post-partum.

#### Birth spacing and family planning

Knowledge of increased risk associated with a birth-to-pregnancy interval of < 24 months: Percentage of mothers of children 0- < 24 months of age who could cite at least two reasons why having a birth-to-pregnancy interval of < 24 months is risky.

Current contraceptive use among mothers of young children: Percentage of non-pregnant mothers of children age 0- < 24 months who are using a modern contraceptive method.

#### Breastfeeding and child nutrition

Exclusive breastfeeding (0- < 6 months): Percentage of infants 0- < 6 months of age who were given only breast milk in the previous 24 hours.

Vitamin A supplementation for child: Percentage of children 6- < 24 months of age who received a dose of vitamin A in the previous 6 months (according to the child’s health card or maternal recall).

Infant and young child feeding for children 6- < 24 months of age: Percentage of infants and young children aged 6- < 24 months fed appropriately during the previous 24 h.

#### Acute respiratory infection

Appropriate care seeking for pneumonia: Percentage of children 0- < 24 months of age with chest-related cough and fast and/or difficult breathing in the previous 2 weeks who were taken to an appropriate health provider within 48 h of onset of symptoms.

#### Diarrhea prevention and case management

Use of oral rehydration therapy during a diarrheal episode: Percentage of children 0- < 24 months of age with diarrhea in the previous 2 weeks who received oral rehydration solution and/or recommended home fluids.

Increased fluid intake during a diarrheal episode: Percentage of children 0- < 24 months of age with diarrhea in the previous 2 weeks who were offered more fluids than usual during the illness.

Increased food intake during a diarrheal episode: Percentage of children 0- < 24 months of age with diarrhea in the previous 2 weeks who were offered the same amount or more food than usual during the illness.

Zinc treatment for diarrhea: Percentage of children 0- < 24 months of age with diarrhea in the previous 2 weeks who were treated with zinc supplements.

#### Childhood immunization

Measles immunization: Percentage of children 12- < 24 months of age who had received a measles vaccination, as verified on the child health card.

Vaccination coverage: Percentage of children 12- < 24 months of age who had received all required antigens and doses at the time of the survey (BCG, PENTA 1–3, polio 1–3, and measles), as verified on the child health card.

#### WASH (water, sanitation and hygiene)

Regular point of use water treatment: Percentage of households with children 0- < 24 months of age that treat water effectively and regularly.

Safe water storage: Percentage of households with children 0- < 24 months of age that store water safely.

Safe feces disposal: Percentage of households with children 0- < 24 months of age that disposed of the youngest child’s feces safely the last time s/he passed stool.

Hand washing at critical times: Percentage of mothers of children 0- < 24 months of age who washed their hands with soap before food preparation, before feeding children, after defecation, and after attending to a child who had defecated.

Appropriate hand washing station: Percentage of mothers of children 0- < 24 months of age who lived in households with a designated place for hand washing with soap and water.

## Abbreviations

AMTSL: Active management of the third stage of labor
BCG: Bacillus Calmette-Guérin (vaccine against tuberculosis)
CBIO: Census-Based, Impact-Oriented Approach
ENC: Essential Newborn Care
KPC: Knowledge, practice, and coverage (household survey)
MSPAS: *Ministerio de Salud Pública y Asistencia Social* (Ministry of Public Health and Social Assistance)
PENTA: Pentavalent vaccine (diphtheria, pertussis, tetanus, Haemophilus influenza type B, and Hepatitis B)
TT: Tetanus toxoid (vaccine)
USAID: United States Agency for International Development

## Footnotes

1 These municipalities are districts and are located in the Department (state) of Huehuetenango.

2 The administration of vitamin A to mothers during the first 2 months postpartum was a government policy during the period of Project implementation, but it was later discontinued.
